# Primed Infusion with Delayed Equilibrium of Gd.DTPA for Enhanced Imaging of Small Pulmonary Metastases

**DOI:** 10.1371/journal.pone.0054903

**Published:** 2013-01-31

**Authors:** Tammy L. Kalber, Adrienne E. Campbell-Washburn, Bernard M. Siow, Elizabeth Sage, Anthony N. Price, Katherine L. Ordidge, Simon Walker-Samuel, Sam M. Janes, Mark F. Lythgoe

**Affiliations:** 1 UCL Centre of Advanced Biomedical Imaging, Division of Medicine and Institute of Child Health, University College London, London, United Kingdom; 2 Centre for Respiratory Research, Department of Medicine, University College London, London, United Kingdom; Cincinnati Children’s Hospital Medical Center, United States of America

## Abstract

**Objectives:**

To use primed infusions of the magnetic resonance imaging (MRI) contrast agent Gd.DTPA (Magnevist), to achieve an equilibrium between blood and tissue (eqMRI). This may increase tumor Gd concentrations as a novel cancer imaging methodology for the enhancement of small tumor nodules within the low signal-to-noise background of the lung.

**Methods:**

A primed infusion with a delay before equilibrium (eqMRI) of the Gd(III) chelator Gd.DTPA, via the intraperitoneal route, was used to evaluate gadolinium tumor enhancement as a function of a bolus injection, which is applied routinely in the clinic, compared to gadolinium maintained at equilibrium. A double gated (respiration and cardiac) spin-echo sequence at 9.4T was used to evaluate whole lungs pre contrast and then at 15 (representative of bolus enhancement), 25 and 35 minutes (representative of eqMRI). This was carried out in two lung metastasis models representative of high and low tumor cell seeding. Lungs containing discrete tumor nodes where inflation fixed and taken for haematoxylin and eosin staining as well as CD34 staining for correlation to MRI.

**Results:**

We demonstrate that sustained Gd enhancement, afforded by Gd equilibrium, increases the detection of pulmonary metastases compared to bolus enhancement and those tumors which enhance at equilibrium are sub-millimetre in size (<0.7 mm^2^) with a similar morphology to early bronchoalveolar cell carcinomas.

**Conclusion:**

As Gd-chelates are routinely used in the clinic for detecting tumors by MRI, this methodology is readily transferable to the clinic and advances MRI as a methodology for the detection of small pulmonary tumors.

## Introduction

Lung cancer is the commonest cause of cancer death worldwide with 152,000 deaths each year in the US (NCI 2011). Accurate and cost effective screening methods for lung cancer are desperately needed. Screening should be sensitive to small nodules, specific to the disease, able to detect suitability of patients for radical treatments and be non-invasive. Prior screening trials with chest radiographs have not found a reduction in lung cancer mortality [1], [2], [3]. Computer tomography (CT) is more sensitive than chest radiography and has recently been shown to be effective at reducing lung cancer mortality, although the feasibility and cost effectiveness of mass CT screening is still disputed [4], [5], [6]. CT imaging can detect pulmonary nodules as small as 2 to 3 mm, but current guidelines deem these small lesions clinically insignificant [7]. It also lacks the specificity to distinguish between benign and cancerous tumors, and nodules of 4 to 10 mm are followed up by repeated CT scans thereby increasing radiation burden [8].

Positron emission tomography (PET) with fluorodeoxyglucose is superior to CT in differentiating between malignant and benign tumors [9], [10]. Preoperative use of PET has led to a reduction in the number of unnecessary thoracotomies in patients considered to be operable on the basis of CT criteria [11], [12]. However PET has a low spatial resolution and reduced specificity for nodules smaller than 10 mm [13]. Further, specificity of PET in nodules larger than 10 mm can fall as low as 60% [14].

Magnetic resonance imaging (MRI) offers distinct advantages over both CT and PET as it uses non-ionising radiation and has better anatomical resolution in most tissues [15]. Lung imaging however is challenging for MRI, due to its low tissue density, many air-tissue interfaces, as well as respiratory and cardiac motion artefacts, and thus it has not reached mainstream clinical use [16], [17].

Gadolinium(III) (Gd) chelates have been widely used to detect and investigate functional tumor perfusion by MRI in other organs [18], [19]. Gd-chelates are small molecules with short half lives, which leak out of capillaries into the interstitial space. Tumor blood vessels are a chaotic network of thin-walled leaky vessels leading to increased accumulation and retardation of wash out of Gd within viable tumor tissue termed the enhanced permeability and retention (EPR) effect [20], [21]. As Gd-chelates are normally given as a single bolus, poorly vascularised tumors such as very small pre-angiogenic tumors may have limited enhancement [22]. However, studies combining bolus with subsequent infusion show that Gd concentrations in tissues of differing permeabilities can be increased [23]. Although the use of gadolinium for dynamic contrast enhancement (DCE) has had limited use in lung tumor imaging [24], it is conceivable that using a primed infusion, an equilibrium of Gd concentration between the vasculature and the interstitial spaces can be reached and sustained. This technique has been termed eqMRI and has been utilised to image myocardial fibrosis in humans [25], as well as systemic amyloidosis in preclinical murine models [26]. We hypothesised this technique may therefore improve the imaging of small lung tumors where equilibrium can provide continuous high concentrations of Gd enhancement.

In this study, primed infusions with a delay before equilibrium (eqMRI) of the Gd(III) chelator Gd.DTPA (Magnevist) were used to evaluate gadolinium tumor enhancement as a function of a bolus injection, which is applied routinely in the clinic, compared to gadolinium maintained at equilibrium. This was carried out in two lung metastasis models representative of high and low tumor cell seeding. We demonstrate that eqMRI specifically improves the detection of small pulmonary metastases compared to bolus enhancement. This methodology would be readily transferable to the clinic using a similar strategy to that used by Flett A.S. et al. in humans [25] and advances MRI as a methodology for the detection of small primary or metastatic tumors.

## Materials and Methods

### Tissue Culture

Cells were grown in T175 flasks (Fisher Scientific, Loughborough, UK) in Dulbecco’s Modified Eagles Medium (DMEM) (Invitrogen, Paisley, UK), supplemented with 10% heat inactivated fetal calf serum (GIBCO, Grand Island NY, USA) in a humidified incubator at 37°C with 95% air, and 5% CO_2_.

### Lentiviral Production and Cell Transduction

Lentivirus vectors pseudotyped with the vesicular stomatitis G protein (VSV-G) were generated by transfection into 293T human kidney cells (ATCC) of a packaging construct (pCpr-ENV), a plasmid producing the VSV-G envelope (pVSV-G) and the vector encoding yellow fluorescent protein (YFP) and the luciferase gene (*Luc)* (pLIONII-HYG-Luc2YFP) as described previously [27]. Plasmid vectors were kindly provided by Dr S Goldie (Cancer Research Institute, CRUK, Cambridge UK). Conditioned medium was harvested at 24 hours and passed through 0.45 mm filters. The transduction of non-confluent MDA-MB-231 cells (CRUK) in a T175 flask was performed using 4 mg/ml polybrene (20 µl polybrene with 20 µl virus in 20 ml medium). The lentivirus was left for 48 hours before changing media. 24 hours later the presence of YFP was assessed by fluorescent microscopy. As the luciferase lentivirus contains a hygromycin resistance gene, 200 µg/ml Hygromycin B was added to culture media for 48 hours. Cells were found to be 96% transfected by flow cytometry using a FACS Calibur (Becton Dickinson, Franklin Lakes NJ, USA).

### Animal Model

All animal studies were approved by the University College London Biological Services Ethical Review Committee and licensed under the UK Home Office regulations and the Guidance for the Operation of Animals (Scientific Procedures) Act 1986 (Home Office, London, United Kingdom). Animal weights were recorded every two days and tumor monitoring was carried out weekly by bioluminescence and MRI scanning with animal monitoring equipment. All procedures were carried out under breathing anaesthesia. Mice were sacrificed with 200 µl pentobarbital by intraperitoneal (i.p) injection (200 mg/ml) and death was confirmed by exsanguination prior to lung inflation (see histology). Male NOD SCID gamma (NSG) mice, 6–8 weeks, were anaesthetised using 2% isoflurane and the tail vein cannulated for infusion of cells. Mice either received a high seeding of cells (2×10^6^ cells in 200 µl serum free media, n = 6, 3 eqMRI & 3 high dose) to achieve rapid growing multiple metastases for weekly longitudinal evaluation, or a low seeding of cells (1×10^5^ cells in 200 µl serum free media, n = 3) to produce slower growing discrete nodules. These low seeding tumors were taken for histology as soon as they were identified by MRI. Control mice received no cells. Mice were imaged using bioluminescence directly after injection and then by both bioluminescence and MRI at weekly time points.

### Bioluminescence Imaging

Mice were anesthetized with 2% isoflurane before and during imaging. An aqueous solution of D-luciferin (Regis Technologies Inc, Morton Grove, IL, USA) at 150 mg/kg in a volume of 200 µl was injected i.p 10 minutes before imaging. Imaging was performed using an IVIS® Lumina II bioluminescent scanner (Caliper Life Sciences, Hopkinton, MA, USA). Quantification of the bioluminescent signal was performed using a region of interest (ROI) covering the whole thorax using Living Image software (version 4: Caliper Life Sciences).

### eqMRI

**Figure 1:**
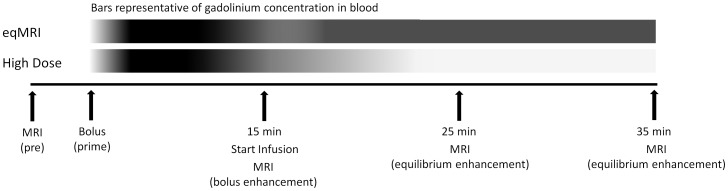
Experimental plan showing timings of bolus and infusion as well as scan times. The bars above are an assumed grey scale representation of gadolinium concentration within the blood where black is high and white low concentration.

Mice were anesthetized with 2% isoflurane before and during imaging. Two i.p lines were used to administer either a bolus or an infusion of Gd.DTPA (Magnevist®, Schering, Berlin). Although, the kinetics of intravenous and i.p injection is different once equilibrium is reached the steady state is the same [26]. For this purpose the i.p route is more suitable and the dosing has been based on the late Gd i.p mouse dose described by Price A.N *et al.*2011 [28] with the subsequent infusion half the bolus dose over 1 hour as described for eqMRI by Campbell A.E *et al.* 2011 [26]. Imaging was performed on an Agilent 9.4T scanner (Agilent Technologies. Santa Clara, CA, USA) with a 39 mm coil (RAPID Biomed, Rimpar, Germany) using a multi-slice double gated (respiration and cardiac) spin-echo sequence with the following parameters: TR ∼ 3.6 s (between RF TR 120–150 ms) determined by gating and number of slices (breathing rate ∼ 50 breaths/minute, and heart rate 400–500 beats/minute), TE = 9.6 ms, FOV = 30×30 mm (3×3 cm), averages = 1: matrix size = 256×128, slices = 10, thickness = 1 mm. Scan time ∼ 8 minutes. After the initial pre contrast scan, mice were given a bolus of 0.6 mmol/kg Gd.DTPA (prime) and after 15 minutes an infusion of 0.005 mmol/kg/min Gd.DTPA was started. Images were acquired directly after the infusion was started at 15 minutes, synonymous with tumor enhancement from the bolus only (preliminary bolus experiments showed this to be the maximal enhancement in subcutaneous tumors via i.p). Images were acquired again at both 25 and 35 minutes, equivalent to tumor enhancement caused by Gd equilibrium in normal tissues (Figure 1). This methodology of bolus with delayed infusion allows for direct comparison of tumor enhancement following bolus compared to equilibrium.

### eqMRI vs High Dose

To assess if tumor enhancement at 25 minutes onwards was due to Gd equilibrium and not just the increased dose of Gd, the total dose of Gd.DTPA for eqMRI was given as a single bolus dose (high dose) in three mice at day 14 (high tumor seeding mice) and compared to eqMRI at the same imaging time points (Figure 1).

### MRI Data Analysis

#### Signal-to-noise (Figure S1)

All images were analysed using ImageJ (Rasband, ImageJ, U.S. National Institutes of Health, Bethesda, Maryland, USA, http://rsb.info.nih.gov/ij/, 1997–2008). Signal-to-noise ratios (SNR) for control lung were obtained from a ROI from whole lung (excluding major vessels) at each time point. To assess the enhancement in individual tumors, ROIs for SNR were drawn around a discrete lesion at the first point of tumor visualisation and then loaded onto the corresponding pre or post images to give the SNR time course for each tumor. Tumors were classed into two; either visible (detected without Gd) or enhancing (detectable after Gd only). In the low tumor cell seeding model, each mouse had one enhancing tumor (n = 3) and at least one visible tumor (n = 5) and a two-sample equal variance t-test was used. Whereas, in the high tumor cell seeding model, three enhancing and three visible tumors were chosen from each mouse (n = 9). Therefore a two-tailed paired t-test assuming equal variances was performed at each time point to determine significant difference, at the 5% level statistical significance.

#### Tumor volume measurements (Figure S2)

To assess the increase in visualized tumors by Gd enhancement, an ROI was drawn around the whole lung (excluding major vessels) on each slice and the total lung volume calculated. As the signal intensity (SI) values of control lung were low, SI values above this threshold were deemed as either tumor or normal enhancing tissue. A threshold was applied from the highest SI value of control lung and the volume of the remaining tissue calculated as a percentage of the total lung volume. To account for the normal enhancing tissue the value obtained from control lung was subtracted from this as a correction value. The resulting volume left is therefore that of tumor and is termed the “percentage lung tumor volume”. To assess if Gd equilibrium increased the number of tumors visible compared to bolus, the percentage lung tumor volume at 15, 25 and 35 minutes was divided by the pre contrast volume and expressed as a percentage increase, termed as the “percentage increase in observed tumors”. A similar approach to assessing lung tumor burden has been described by Tidwell V.K *et al.* 2012 [29]. A two-tailed paired t-test assuming equal variances was performed at each time point to determine significant difference (n = 3 per group), at the 5% level statistical significance. Errors are given as standard errors of the mean (s.e.m) for both SNR and the percentage increase in observed tumors.

### Histology

Inflated lungs were fixed in 4% formalin for 4 hours at 4°C and then incubated overnight in 15% sucrose in PBS at 4°C. Tissue was dehydrated in 70% ethanol and embedded in paraffin. Sections corresponding to MR images were cut at 5 µm. Sections were stained with either Haematoxylin (Sigma-Aldrich, Dorset, UK) for 1 minute and Eosin (Sigma-Aldrich, UK) for 30 seconds (H & E), or by CD34 immunohistochemistry. Immunohistochemistry, including antigen retrieval and signal amplification was carried out as previously described [30]. Antibodies for CD34 detection were as follows; rat anti-mouse primary CD34 antibody at a 1∶5 dilution (Hycult Biotech, The Netherlands), biotinylated secondary antibody at a 1∶200 dilution (Dako, Denmark). Immunoreactions were visualized using diaminobenzidine hydrochloride (DAB Substrate Kit, Vector Laboratories) as chromogen. Sections were counterstained with haematoxylin as above. Images were obtained using an Olympus BX40 microscope (Olympus Imaging, Essex, UK) with Qcapture software (QImaging, Canada). Composite images of whole lungs were made from multiple single 100x images using Adobe Photoshop CS (Adobe Systems Inc, California, USA). Tumors were sized by histology at the widest midpoint on H & E stained slides by Qcapture software rather than by MRI due to partial volume and susceptibility effects that can over estimate tumor size.

## Results

**Figure 2:**
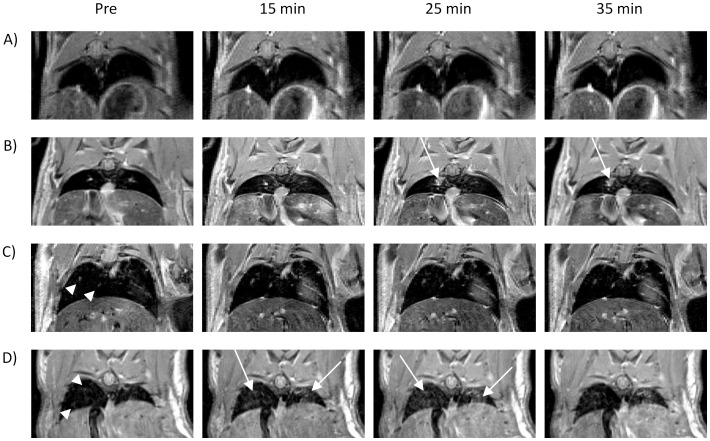
MR images over the 35 minute eqMRI time course. A) control lungs, B) a discrete gadolinium enhancing tumor nodule (25 minutes) (low tumor cell seeding model), C) tumor nodules visible on the pre contrast scan (low tumor cell seeding model), D) tumor presentation representative of a high tumor cell seeding model lung at day 14. Tumors visible on pre contrast (white arrowheads) and tumors only visible with gadolinium enhancement (white arrows) are indicated.

Two models of lung metastasis representative of high and low tumor cell seeding were evaluated by eqMRI (Figure 1, experimental dosing plan). Animals in the high tumor cell seeding model were followed weekly longitudinally up until day 28, these animals appeared healthy and recovered well at all time points after imaging (n = 3). Animals receiving low tumor cell seeding, generating discrete tumor nodules were taken for histology as soon as they were identified by MRI (n = 3). Bioluminescence confirmed tumor growth in both models prior to identification by MRI (Figures S3 and S4). The SNR was used to assess the enhancement in individual tumors, while the increase in the volume of tumors becoming visible after Gd enhancement was expressed as the “percentage increase in observed tumors”. Control lungs did not show any contrast enhancement throughout eqMRI (Figure 2A) (SNR was 1.68±0.57 at pre contrast and constant throughout p = 0.25, n = 3).

### Gd.DTPA Sustained at Equilibrium Improves the Detection of Pulmonary Tumors Compared to Bolus Enhancement

**Figure 3:**
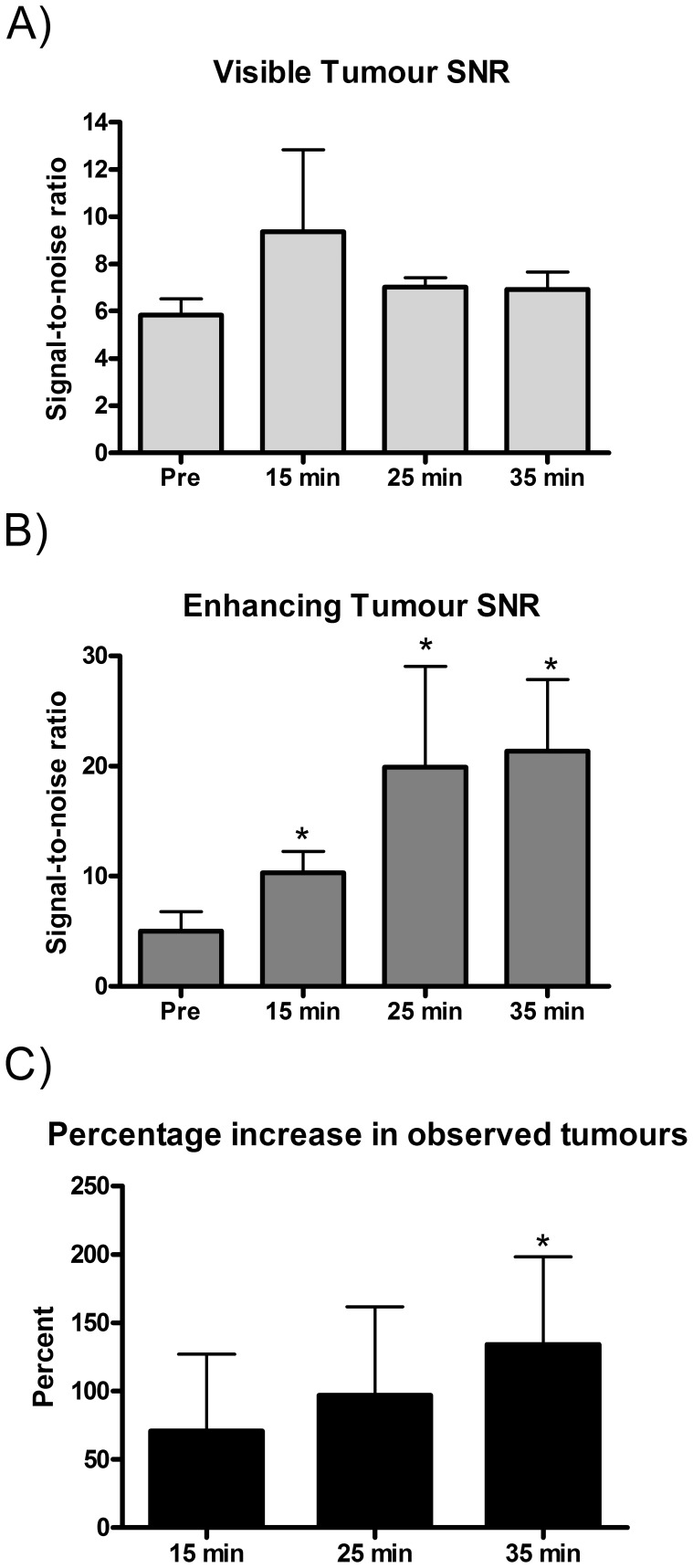
Low tumor cell seeding model mean values for signal-to noise (SNR) and the percentage increase in observed tumours for eqMRI. A) SNR of tumors already visible on pre contrast images, B) SNR of tumors that were visible due to Gd enhancement from 15 minutes onwards, C) the percentage increase in observed tumors by Gd enhancement at each time point compared to pre contrast. Values are mean ± s.e.m. * p = <0.05 from pre (A and B) or from 15 min (C).

Lung metastases were detectable in animals receiving high tumor cell seeding as areas of hyperintensity from day 14, and from day 21 in low tumor cell seeding animals. eqMRI imaging defined two sets of tumors. Tumors visible on pre contrast images that did not change in presentation and their SNR values remained constant at all time points are termed as visible tumors (Figure 2 white arrowheads, Figure 3A). Tumors that only became visible with Gd enhancement at 15, 25 or 35 minutes but were not apparent on the pre scan image are termed enhancing tumors (Figure 2 white arrows). All high tumor cell seeding mice presented with multiple visible and enhancing tumors (n = 9 each). Whereas, low tumor cell seeding mice exhibited at least one visible (n = 5) and one enhancing tumor (n = 3) by MRI prior to histology. As the mice in the low tumor cell seeding model were taken as soon as tumors were visible by MRI for histology, this limited the numbers of tumors per mouse that could be assessed. Although the trends in Gd enhancement are similar for both models this needs to be taken into account. Also, the number of mice per group (n = 3) is a limiting factor when evaluating the percentage increase in observed tumor data.

**Figure 4:**
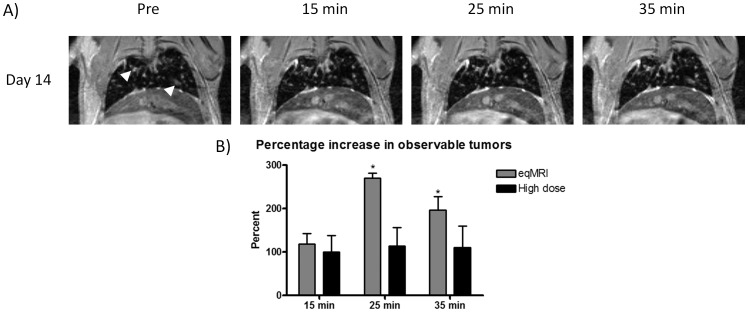
MR images of a high tumor cell seeding model mouse at day 14 over the 35 minutes for high dose Gd.DTPA (white arrowsheads denote visible tumors) (A), and the corresponding mean values for the percentage increase in observable tumors compared to that of high tumor cell seeding mice at day 14 for eqMRI (B). Values are mean ± s.e.m. * p = <0.05 from high dose.

Although a few diffuse tumors become evident at 15 minutes (Figure 2D 15 minutes, arrows) after the initial bolus prime, the majority of enhancing tumors were observed due to sustaining Gd equilibrium at 25 minutes onwards (Figure 2B and D, arrows). This is also evident in the rise in SNR in individual tumors from pre contrast compared to 15 minutes (p = <0.05) with a further increase at 25 minutes which was sustained at 35 minutes (p = <0.05) (Figure 3B and Figure S5B). eqMRI resulted in an significant increase in the percentage observed tumors at Gd equilibrium compared to bolus (p = <0.05) (Figure 3C and Figure S5C). Although in the low tumor cell seeding model there is an increase in the percentage observed tumors at 35 minutes compared to 25 minutes (Figure 3C), for the high tumor cell seeding model there is a reduction at 35 minutes compared to 25 minutes (Figure 4C & Figure S5C). This is most likely due to the amount of Gd accumulating within the tumors leading to susceptibility artefacts and T_2_* affects, which is more apparent in high tumor cell seeding model.

### Gd.DTPA at Equilibrium not the Dose Improves Pulmonary Tumor Detection

When a bolus dose of Gd.DTPA equivalent to the total dose of Gd.DTPA used in eqMRI was given as single bolus, animals did not present late enhancing tumors at 25 or 35 minutes (Figure 4A). Tumors visible on the pre contrast remained unchanged as in eqMRI (Figure 4A, white arrows). The percentage increase in observed tumors for both eqMRI and high dose Gd.DTPA was the same at 15 minutes (Figure 4B). Although, the high dose would be expected to increase the percentage observed tumors compared to the lower dose used in eqMRI at this time point, there appears to be no effect. This may be due to intergroup heterogeneity, but as fewer enhancing tumors appear to be present in the high dose this may indicate that any enhancing tumors are saturated with Gd causing susceptibility and T_2_* effects as described previously affecting visualization. As the bolus has a limited time window for enhancement compared to Gd at equilibrium there is no further enhancement at 25 and 35 minutes for the high dose and the percentage increase in observed tumors is therefore significantly higher for eqMRI than the high bolus dose at these time points, confirming the contribution of Gd equilibrium to lung tumor enhancement (Figure 4B).

### Gd Equilibrium Enhancing Tumors are Sub-millimeter in Size

**Figure 5:**
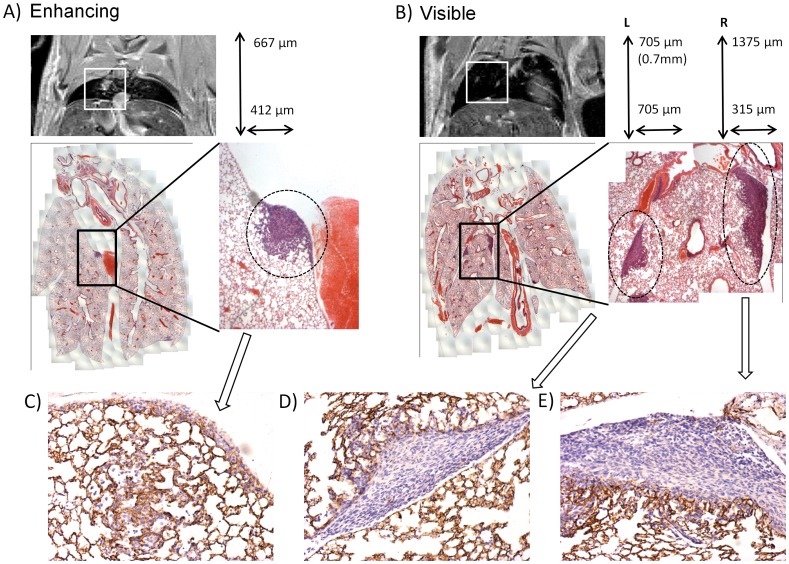
An MR image of a low tumor cell seeding model mouse lung the corresponding whole lung composite H & E stained section, a blown up area and sizing of tumor (white box), and CD34 immunohistochemistry image. A) a single enhancing tumour (at 25 minutes) with corresponding CD34 image (C), B) two visible tumors (pre contrast image) with corresponding CD34 images left (D) and right (E).

Histology examination of lung sections corresponding to MR images for low tumor cell seeding mice, showed that tumors enhancing at 25 minutes onwards due to Gd equilibrium were sub-millimeter (<0.7 mm^2^) tumors (Figure 5A), whereas tumors visible on pre contrast images tended to be larger (Figure 5B). Occasionally, visible tumors were also sub-millimeter in size and histology showed these as solid tumors (Figure 5D and E). Enhancing tumors however, appeared to line and fill the avelolar spaces with similar morphology to that shown by early bronchoavelolar cell carcinomas and atypical adenomatous hyperplasia (Figure 5C). Longitudinal evaluation of enhancing tumors showed that with tumor growth they become visible on the pre contrast scan further confirming them as tumors (Figure S6 white arrow at day 14 and subsequent white arrow head at day 21).

## Discussion

In this study, primed infusions with a delay in equilibrium (eqMRI) of Gd.DTPA were used to compare gadolinium tumor enhancement of pulmonary tumors after bolus (as routinely used in the clinic), or gadolinium maintained at equilibrium. The results show that the maximal enhancement of pulmonary tumors was due to sustained Gd enhancement afforded by Gd equilibrium (25 minutes onwards) in both lung metastasis models. As few tumors enhanced after the high dose bolus prime (15 minutes), without Gd equilibrium the majority of enhancing tumors in this study would have been missed with a traditional Gd bolus MRI protocol.

Histological examination confirmed that the Gd equilbrium enhancing tumors were small (<0.7 mm) disorganised tumors with a low cellularity. The tumor cells line and fill the avelolar spaces in a similar manner to early bronchoavelolar cell carcinomas. The low cellular density means that they are not evident on the pre contrast scan and as the tumors are integrated within the avelolar spaces this increases the degree of diffusion of Gd.DTPA into the tumor interstitial spaces [31], [32], [33], [34]. Gd equilibrium is therefore able to increase the concentration of Gd sufficiently, making them visible by MRI.

Both lung metastasis models presented with tumors that were already visible on pre contrast images. These tumors showed no significant contrast enhancement with Gd. Although, histological examination showed that they are generally larger than enhancing tumors, visible tumors could still be sub-millimeter in size. The main difference, except for size, appears to be that they have a higher cell density than the enhancing tumors, which may account for the early visualisation on MRI.

A previous study by Garbow *et al.* used a similar breathing gated sequence and detected lung tumor nodules in mice at a comparable size to the small visible tumors shown in this study [35]. Although the study did not investigate Gd enhancement, it verifies the use of MRI in the detection of sub-millimetre metastases. Other methods for lung metastasis imaging utilizing MRI such as Ultra-short echo time (UTE) imaging [36], [37], [38], [39], fluorinated [40] or paramagnetic molecular oxygen [41] and most recently hyperpolarised ^129^Xe and ^3^H gas [42], [43], [44], [45] have also been developed. A recent study by Branca *et al.* has combined hyperpolarized gas with tumor targeted iron oxide particles enhancing the detection of tumors at sub-millimetre sizes [46].

The gadolinium doses used in this study have been designed for the preclinical identification of small pulmonary metastases in mouse models of lung metastasis due to Gd given as a bolus or Gd maintained at equilibrium. The doses are therefore much higher than what would be used in the clinic. Although, this is a limitation, gadolinium can be maintained at equilibrium in humans at much lower doses and eqMRI has already been utilized in humans to assess myocardial fibrosis [25]. Although the use of this dosing regimen for the imaging of small pulmonary tumors would require further optimization, this methodology has the potential to be a safe and sensitive method for the detection of small primary or metastatic tumors in a similar fashion to CT. The dynamic uptake of Gd-chelates has been shown to improve the discrimination of benign and malignant tumors in other tissues types screening [47]. However, this has not been assessed in this study using eqMRI.

In conclusion, the results for this study demonstrate that a high concentration of sustained Gd enhancement afforded by eqMRI greatly improves the detection of sub-millimetre pulmonary metastases. The diffusion of contrast throughout the tumor mass increases the concentration of Gd retained causing enhancement when Gd equilibrium is reached.

## Supporting Information

Figure S1 **Flow diagram describing how the two sets of tumors were defined and how regions of interests where placed for individual tumor signal-to-noise calculations (see MRI data analysis).** Drawn ROIs are only representative for explanation purposes.
(TIF)

Figure S2 **Flow diagram describing how tumor volumes were calculated to assess the percentage increase in observed tumors due to Gd enhancement (see MRI data analysis).**
(TIF)

Figure S3 **Bioluminescent images of a high tumor cell seeding model mouse directly after infusion and at days 7 and 14 (A) and the corresponding plot of increasing bioluminescent signal (B) consistent with tumor growth.**
(TIF)

Figure S4 **Bioluminescent images of a low tumor cell seeding model mouse directly after infusion and weekly to 35 days (A) and the corresponding plot of increasing bioluminescent signal (B) consistent with tumor growth.**
(TIF)

Figure S5 **High tumor cell seeding model weekly mean values for signal-to noise (SNR) and the percentage increase in observed tumours for eqMRI.** A) SNR of tumors already visible on pre contrast images, B) SNR of tumors that were visible due to Gd enhancement from 15 minutes onwards, C) the percentage increase in observed tumors by Gd enhancement at each time point compared to pre contrast. Values are mean ± s.e.m. * p = <0.05 from pre (A and B) or from 15 min (C).
(TIF)

Figure S6 **MR images over the 35 minute eqMRI time course for a high tumor cell seeding model mouse at day 14 (top row), and day 21 (bottom row).** Tumors that enhance due to gadolinium equilibrium (white arrow) at day 14, subsequently due to tumor growth are then visible on the pre contrast image (white arrowheads) at day 21.
(TIF)
