# Supplementary figures and images for: Primed Infusion with Delayed Equilibrium of Gd.DTPA for Enhanced Imaging of Small Pulmonary Metastases
Source: PLoS One. 2013 Jan 31;8(1):e54903. doi: 10.1371/journal.pone.0054903 (PMC3561448; doi:10.1371/journal.pone.0054903)

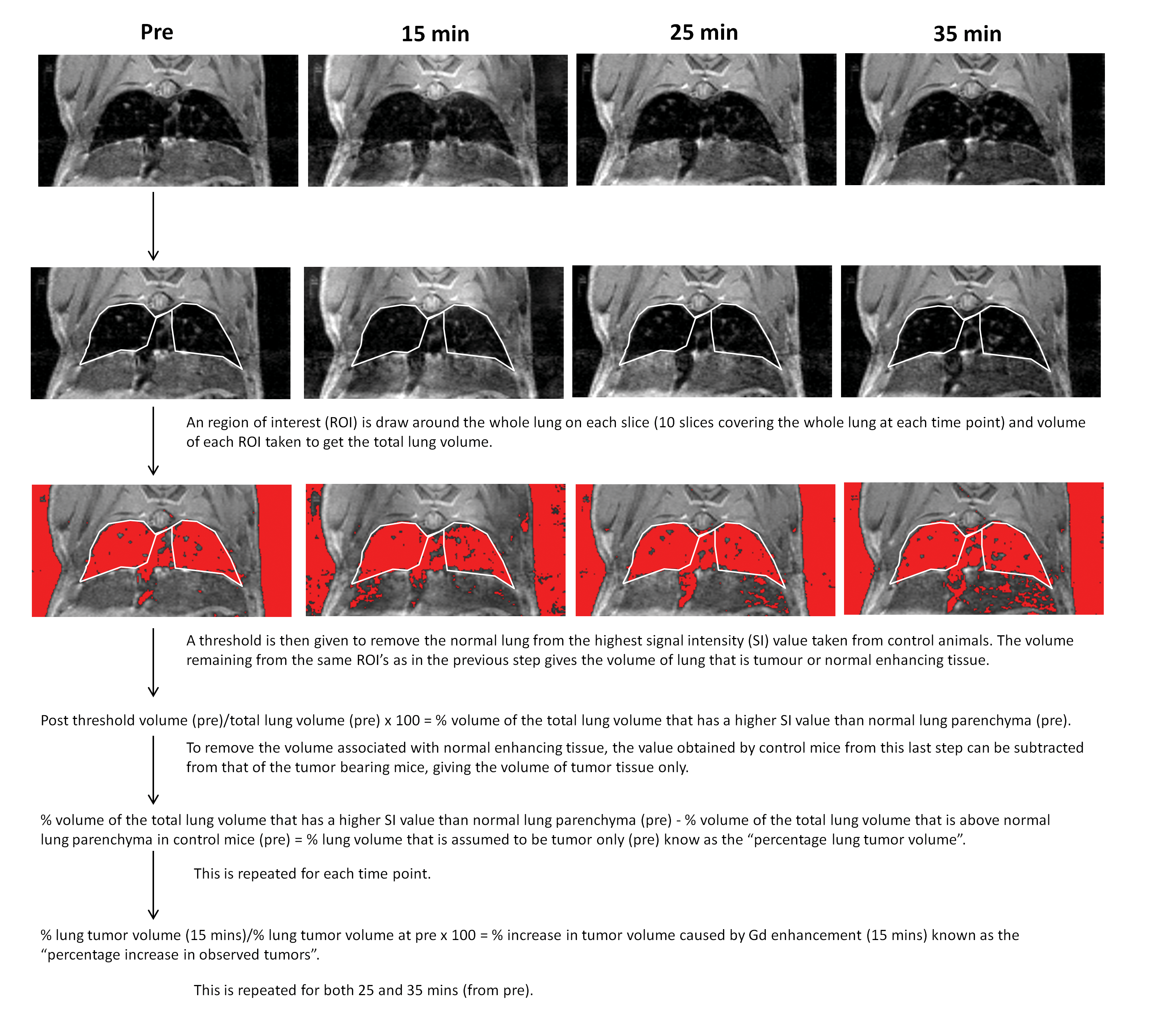

Supplement: Figure S2 — Flow diagram describing how tumor volumes were calculated to assess the percentage increase in observed tumors due to Gd enhancement (see MRI data analysis). (TIF) [file pone.0054903.s002.tif]

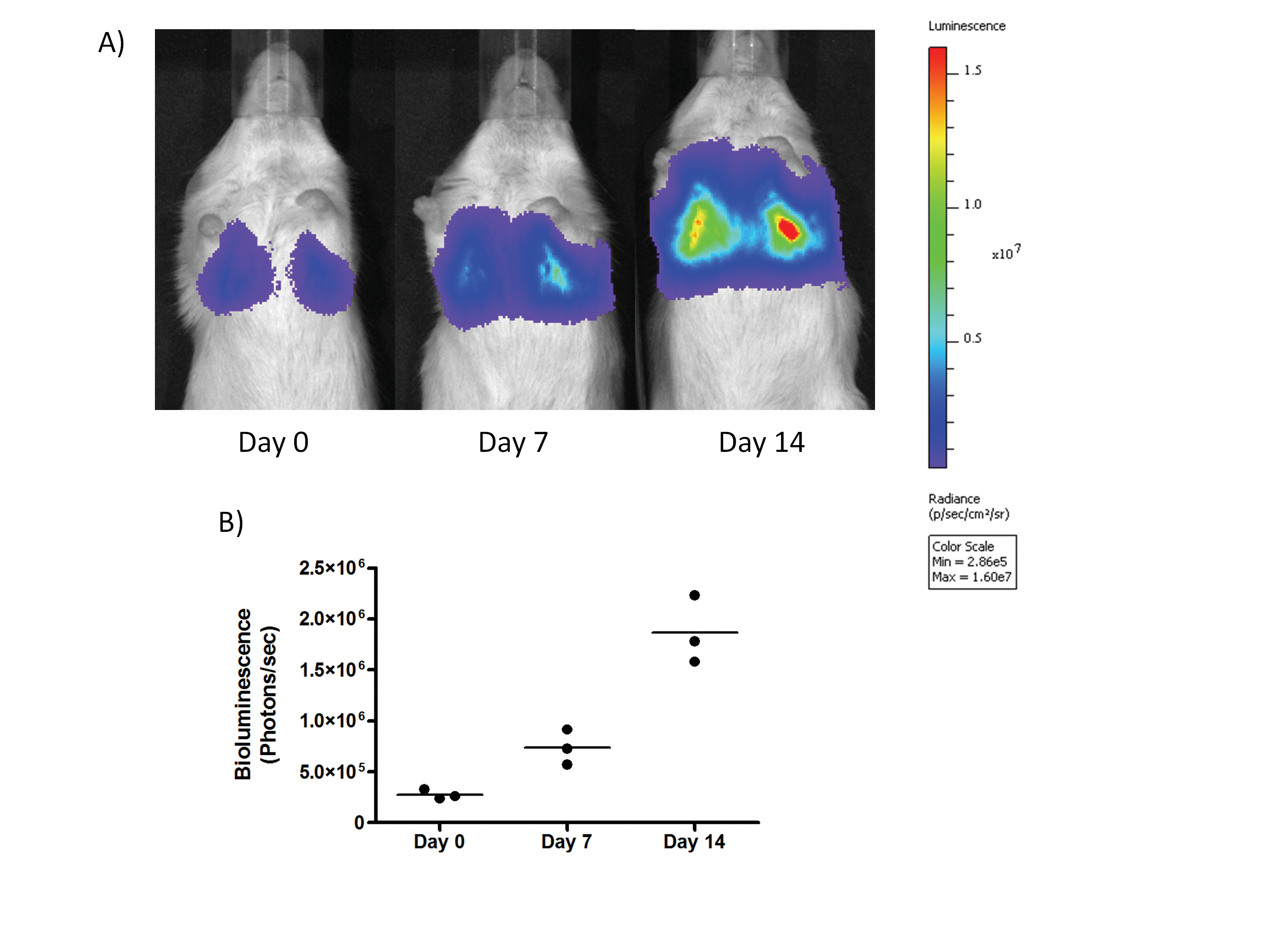

Supplement: Figure S3 — Bioluminescent images of a high tumor cell seeding model mouse directly after infusion and at days 7 and 14 (A) and the corresponding plot of increasing bioluminescent signal (B) consistent with tumor growth. (TIF) [file pone.0054903.s003.tif]

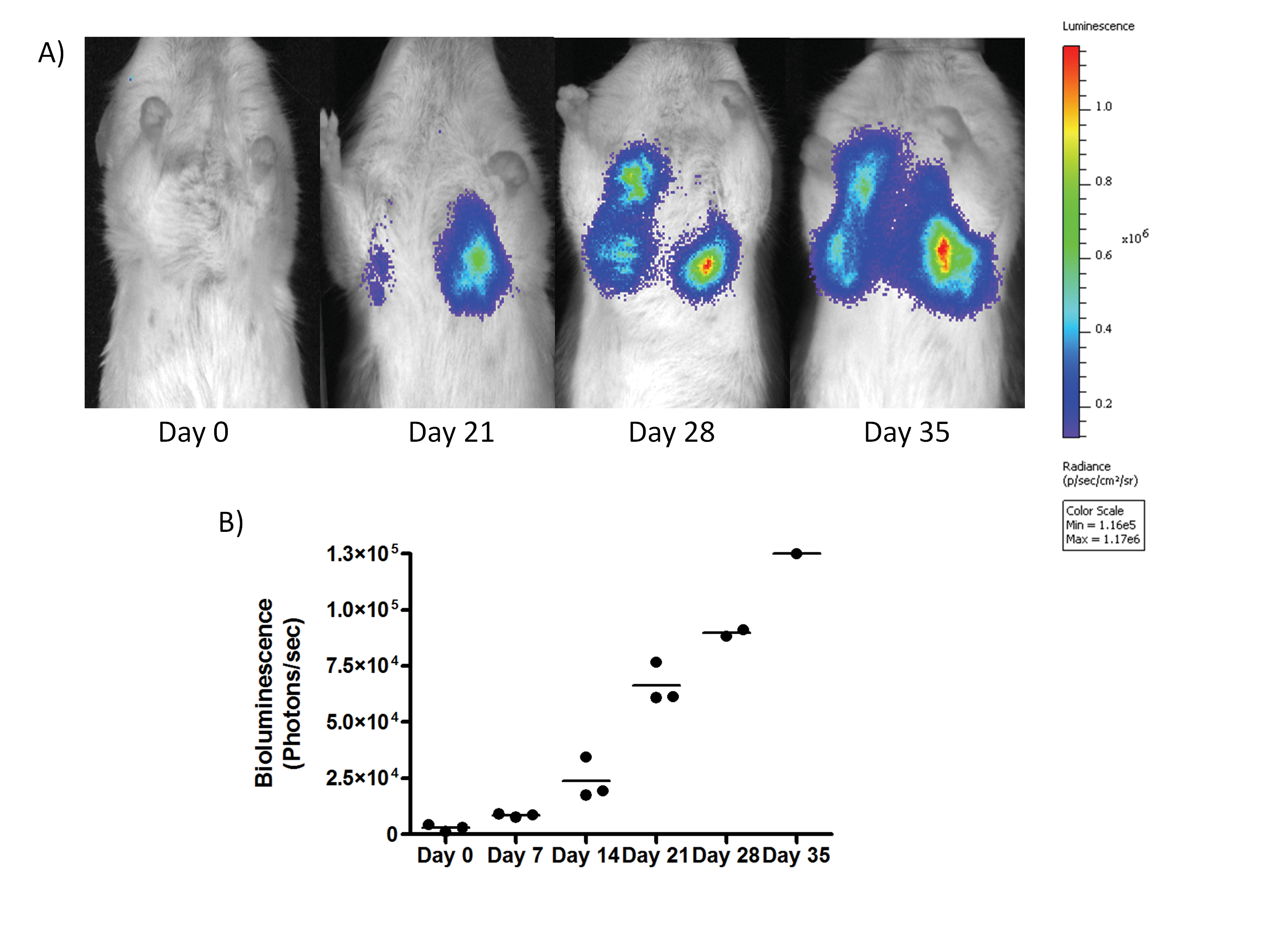

Supplement: Figure S4 — Bioluminescent images of a low tumor cell seeding model mouse directly after infusion and weekly to 35 days (A) and the corresponding plot of increasing bioluminescent signal (B) consistent with tumor growth. (TIF) [file pone.0054903.s004.tif]

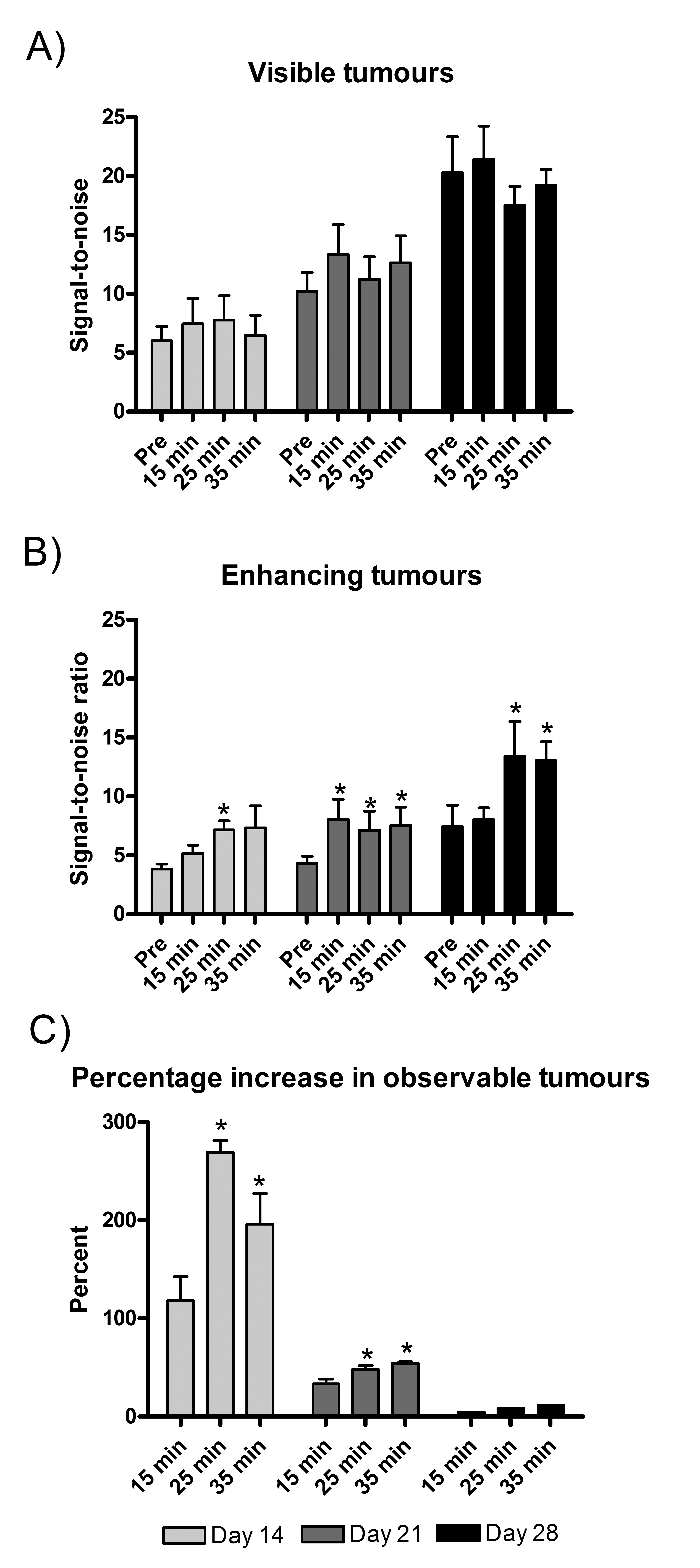

Supplement: Figure S5 — High tumor cell seeding model weekly mean values for signal-to noise (SNR) and the percentage increase in observed tumours for eqMRI. A) SNR of tumors already visible on pre contrast images, B) SNR of tumors that were visible due to Gd enhancement from 15 minutes onwards, C) the percentage increase in observed tumors by Gd enhancement at each time point compared to pre contrast. Values are mean ± s.e.m. * p = <0.05 from pre (A and B) or from 15 min (C). (TIF) [file pone.0054903.s005.tif]

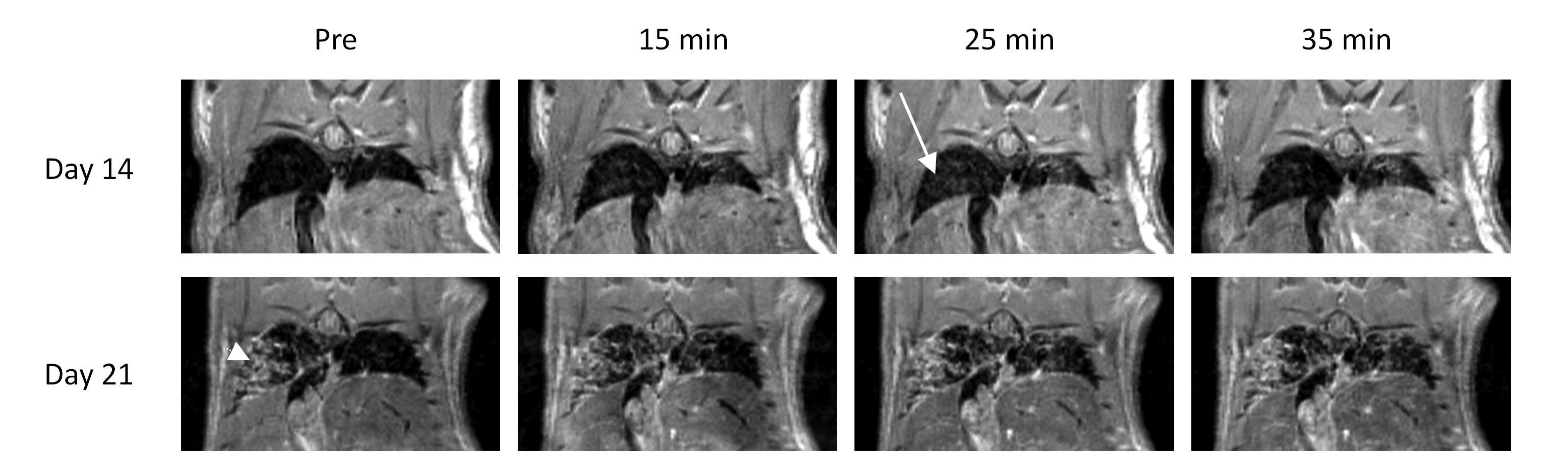

Supplement: Figure S6 — MR images over the 35 minute eqMRI time course for a high tumor cell seeding model mouse at day 14 (top row), and day 21 (bottom row). Tumors that enhance due to gadolinium equilibrium (white arrow) at day 14, subsequently due to tumor growth are then visible on the pre contrast image (white arrowheads) at day 21. (TIF) [file pone.0054903.s006.tif]
